# The Synthesis and Evaluation of Novel Hydroxyl Substituted Chalcone Analogs with *in Vitro* Anti-Free Radicals Pharmacological Activity and *in Vivo* Anti-Oxidation Activity in a Free Radical-Injury Alzheimer’s Model 

**DOI:** 10.3390/molecules18021693

**Published:** 2013-01-28

**Authors:** Ying Pan, Yicun Chen, Qingnan Li, Xiaoyu Yu, Jinzhi Wang, Jinhong Zheng

**Affiliations:** 1 Department of Chemistry, Shantou University Medical College, Shantou 515041, Guangdong, China; 2 Department of Pharmacology, Shantou University Medical College, Shantou 515041, Guangdong, China; 3 Shantou Central Hospital, Shantou 515041, Guangdong, China

**Keywords:** Alzheimer’s, free radical injury, polypheols, hydroxyl-substituted chalcones

## Abstract

Alzheimer’s disease (AD) pathogenesis involves an imbalance between free radical formation and destruction. In order to obtain a novel preclinical anti-AD drug candidate, we synthesized a series of novel hydroxyl chalcone analogs which possessed anti-free radical activity, and screened their effects on scavenging 2,2-diphenyl-1-picrylhydrazyl (DPPH) and OH free radicals *in vitro*. Compound **C7**, 4,2'-dihydroxy-3,5-dimethoxychalcone was found to have potent activity in these anti-free radical activity tests. Further research revealed that **C7** could elevate glutathione peroxidase (GSH-PX) and super oxide dismutase (SOD) levels and lower malonaldehyde (MDA) level *in vivo* in the Alzheimer’s model. The indication of **C7**’s effect on AD needs further study.

## 1. Introduction

Free radicals have been implicated in the etiology of several human diseases, as well as ageing. Many studies indicate that mitochondrial reactive oxygen species (ROS) production and oxidative damage to mitochondrial DNA results in ageing [1,2,3]. Alzheimer’s disease (AD) is the most common form of neurodegenerative disease associated with dementia in elderly people. A growing body of evidence suggests that AD pathogenesis involves an imbalance between free radical formation and destruction [4,5,6,7,8]. This concept originally derived from the free radical hypothesis of aging, with age-related accumulation of free radicals resulting in damaged cell components. That age is a key risk factor in AD provides support for this hypothesis [9,10].

A role for oxidative stress in the pathogenetic cascade of events in AD and other neurodegenerative disorders is appealing because neurons are post-mitotic cells and gradually accumulate oxidative damage over time, which would account for the late life onset and the slowly progressive nature of these disorders [11]. Moreover, ‘antioxidants’ are substances that neutralize free radicals or their actions [12]. Epidemiologic studies show that higher intake of foods with functional attributes including polyphenol, and flavonoid constituents or higher intake of some synthetic products such as vitamin E have a putative antioxidant effects in reducing the risk of AD [13].

**Figure 1:**
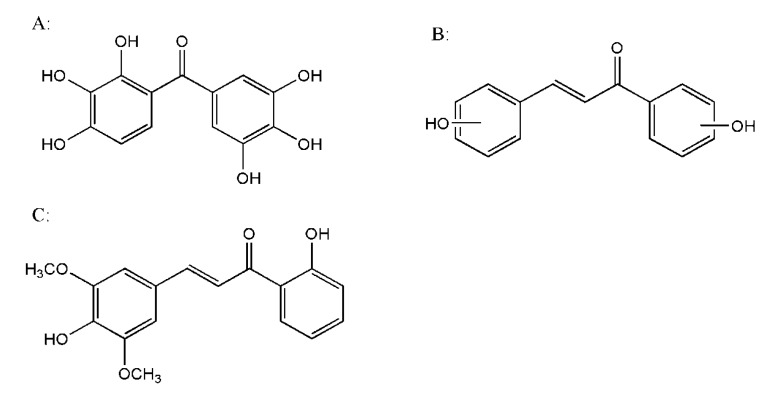
The structure of exifone (**A**); chalcones (**B**); **C7**: the polyphenol-3,5-dimethoxy-4,2'-dihydroxychalcone (**C**).

In the clinic, exifone (Figure 1A) is a classical anti-AD drug that treats cognitive decline associated with aging and corrects memory dysfunction [14]. From the structure of exifone, we can see it is an analogue of the polyhydroxyl chalcones. It was reported that chalcones have a wide spectrum of biological activities, especial antioxidant activity. Hydroxyl chalcones embrace hydroxyl substitutions, one of the key groups to greatly enhance the antioxidant activity of chalcones, mainly because of their easy conversion to phenoxy radicals through the hydrogen atom transfer mechanism [15]. In order to obtain a novel preclinical anti-AD drug candidate, we designed and synthesized the series of compounds **C1**–**7** shown in Table 1 and screened these potential compounds for anti-free radical activity *in vitro* for further study. A preliminary structure-activity relationship study of these compounds was carried out meanwhile. Then, we test the compounds’ effect on SOD, MDA and GSH-PX levels to evaluate the anti-oxidation activity *in vivo* in a free radical-injury Alzheimer’s model.

**Table 1:** Chemical structure, physical properties, ^1^H-NMR and MS spectral data of the synthesized hydroxyl-substituted chalcones.

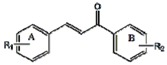
**No.**	**R_1_**	**R_2_**	**Melting point (°C)**	**Physical properties/ Molecular formula**	**^1^H-NMR (400 MHz, DMSO-d_6_)**	**MS( *m/z* )**
**C1**	2-OH	H	76–78	Yellow crystal/	6.89 (1H, d, *J* = 16, =CH)	223.0[M−H]^+^
				C_15_H_12_O_2_	7.29 (1H, d, *J* = 16, =HC)	
					6.89 ~ 8.10 (m, 9H, ArH)	
					10.31 (s, 1H, OH)	
**C2**	H	2'-OH	88–90	Yellow crystal/	7.02 (1H, d,d, *J* = 16, =CH)	223.0[M−H]^+^
				C_15_H_12_O_2_	7.42 (1H, d,d, *J* = 16, =CH)	
					7.02 ~ 8.28 (m, 9H, ArH)	
					12.47(s, 1H, OH)	
**C3**	4-OH	H	83–85	Yellow crystal/	7.75 (1H, d,d, *J* = 15.6, =CH)	223.0[M−H]^+^
				C_15_H_12_O_2_	8.14 (1H, d,d, *J* = 15.6, HC=)	
					6.86 ~ 7.75 (m, 9H, ArH)	
					10.15 (s, 1H, OH)	
**C4**	4-OH	2'-OH	193–195	Transparent crystal/	7.43 (d, *J* = 15.2, 1H, CH= )	239.0[M−H]^+^
				C_15_H_12_O_3_	7.81 (d, *J* = 15.2, 1H, CH= )	
					6.79 ~ 7.80 (m, 8H, ArH)	
					9.58 (s, 1H, OH)	
					11.64 (s, 1H, OH)	
**C5**	4-OH,	2'-OH	158–160	Transparent crystal/	3.77 (s, 3H, OCH3)	269.1[M−H]^+^
	3-OCH_3_			C_15_H_11_NO_5_	7.75 (d, J = 16, 1H, CH= )	
					8.15 (d, J = 16, 1H, CH= )	
					6.78 ~ 7.77 (m, 7H, ArH)	
					9.14 (s, 1H, OH)	
**C6**	4-OH,	2'-OH	144–146	Yellow crystal/	7.56 (d, *J* = 16.4, 1H, CH= )	284.0[M−H]^+^
	3-NO_3_			C_15_H_11_NO_5_	7.97 (d, *J* = 16.4, 1H, CH= )	
					7.02 ~ 8.10 (m, 7H, ArH)	
					11.18 (s, 1H, OH)	
**C7**	4-OH,	2'-OH	172–174	Red crystal/	3.88 (s, 6H, OCH3)	298.9[M−H]^+^
	3,5-2OCH_3_			C_17_H_16_O_5_	7.62 (d, *J* = 16, 1H, CH=)	
					8.02 (d, *J* = 16, 1H, CH=)	
					7.02 ~ 8.35 (m, 6H, ArH)	
					9.20 (s, 1H, OH), 12.91 (s, 1H, OH)	

## 2. Results and Discussion

### 2.1. Synthesis of ***C1**–**7***

**Scheme 1:**
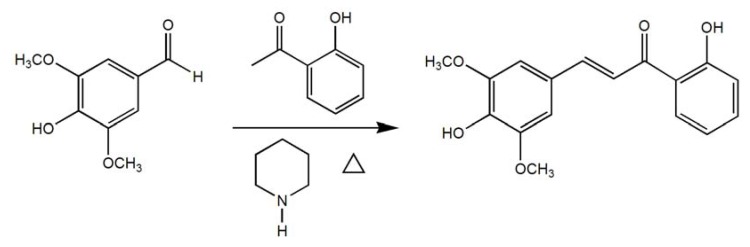
The synthesis of the polyphenol-3, 5-dimethoxy-4, 2'-dihydroxychalcone (**C7**).

It was reported that hydroxyl chalcones had a wide spectrum of biological activities, including antioxidant activity and chalcone analogs such as curcumin, and exifone have anti-AD activity. Hydroxyl-substituted chalcone analogs could develop into novel anti-AD drug candidates. The chalcone analogs **C1**–**7** were successfully synthesized via Claisen-Schmidt condensation using NaOH or pyperidine as a catalyst in this study (Scheme 1). The chemical structures and physical data of the synthesized hydroxyl-substituted chalcones **C1**–**7** were listed in Table 1. ^1^H-NMR and mass spectroscopy identifications of **C1**–**7** were shown in Table 1. Reports indicated hydroxyl substitutions on benzyl rings greatly affected the reactivity. When the total amount of hydroxyl-substitutions (phenolic hydroxyls) on benzaldehydes and acetophenones exceeds one, *i.e.*, easy to ionize in NaOH solution and the carbonyl-C seemed to fail to retain its original polarity, the reaction would fail [16,17], so NaOH is used as a catalyst in the syntheses of **C1**–**3**. In our study, we found piperidine catalysis seems a highly effective and promising method in Claisen-Schmidt condensation for synthesizing dihydroxy-substituted chalcones. Piperidine is successfully used as a catalyst in the syntheses of **C4**–**7**.

### 2.2. Activity of C1–7 in Scavenging DPPH and OH Free Radicals

ROS has been identified as an important mediator of cell structure damage to lipids, proteins, and nucleic acids. ROS has been associated with aging, AD and other neurodegenerative disorders. Host antioxidant defenses control the level of reactive free radicals, but cellular damage occurs when free radicals have overwhelmed this defense. Thus, the antioxidant might be an important therapeutic strategy in inhibiting development of AD and other neurodegenerative disorders.

**Figure 2:**
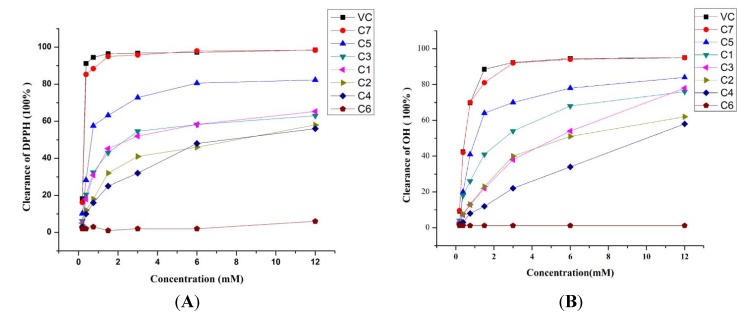
Concentration-clearance on DPPH (**A**), Concentration-clearance on OH (**B**).

Thus, a series hydroxyl substituted chalcone analogs **C1**–**7** were synthesized and their anti-free radical activities were evaluated. Vitamin C, whose anti-oxidation effects were demonstrated in many *in vitro* experiments and in humans and must be ingested for nutrition, was selected as the positive control. After the test processes and statistical calculations, we obtained concentration-clearance (DPPH) curves (Figure 2A). In the same way, concentration-clearance (OH) curve was obtained as follows (Figure 2B). The IC_50_ of VC and **C1**–**7** were listed in Table 2. In this study, results indicated the IC_50_ (OH) and (DPPH) values of **C7** (0.441, 0.255 mM) were similar to that of **VC** (0.442, 0.241 mM) and **C7** showed the strongest anti-free radical activity in all compounds **C1**–**7** *in vitro* (Figure 2, Table 2).

**Table 2:** IC_50_ values for DPPH and OH· clearance of hydroxyl-substituted chalcones.

Compound	OH· clearance IC_50_ (mM)	DPPH· clearance IC_50_ (mM)
C1	1.655	0.836
C2	2.741	1.688
C3	10.040	0.769
C4	5.726	3.65
C5	0.750	0.521
C6	-----	-----
C7	0.441	0.255
Vit C	0.442	0.241

Tests of activities on DPPH and OH assay are one of the best-known and frequently used methods for evaluating the free-radical-scavenging activity. They have been widely used in model systems to investigate the scavenging activities of several natural compounds such as phenolic compounds, anthocyanins, crude mixtures such as methanol extracts of plants, or some synthetic products [11,18].

The study of structure-activity relationships of **C1**–**7** (Table 1) indicated that the effect of **C5**, **C7** on free radical scavenging properties are stronger than **C1**–**4**, and **C6** shows low scavenging capability.

It is not hard to understand that phenolic groups on the A ring and B ring were key to the free-radical scavenging activity. In this study, the results also demonstrated that the OCH_3_ group can increase the free radical scavenging properties (**C5** *vs.* **C1**–**4**). Furthermore, 3,5-dimethoxy can maximize the anti-free radical activity (**C7** *vs.* **C5**). Recently, Chan *et al*. demonstrated that the antioxidant activity of phenolics, such as curcumin is due not only to the number of phenolic groups but also the *ortho*-methoxyphenolic functionality [19]. The *ortho*-methoxy group can form an intramolecular hydrogen bond with the phenolic hydroxy group, making the H-atom abstraction from the *ortho*-methoxyphenols surprisingly easy. Furthermore, the electron withdrawing group NO_2_ in A ring (**C6**) decreased the scavenging properties, but the electron donating group OCH_3_ increases those.

Our comparative study demonstrated that the antioxidant activities of **C1**–**7** can arise from the phenolic groups, *ortho*-methoxyphenol and the electron properties of substituent groups.

### 2.3. Impact of C7 on SOD, MDA and GSH-PX Levels in Scopolamine-Induced AD Model

**Figure 3:**
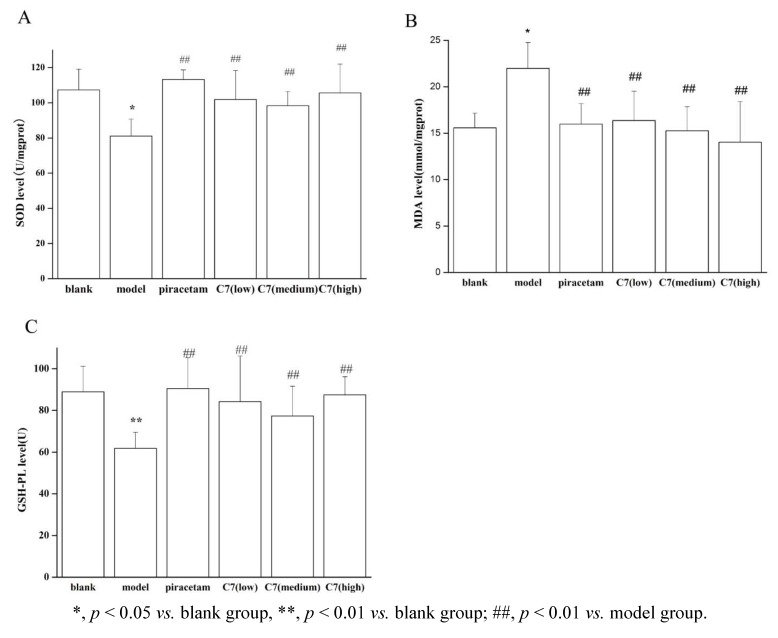
Global cerebral SOD (**A**) MDA (**B**) GSH-PX (**C**) levels for all.

**C7** showed potent anti-oxidation effects, so it was elected for further study on the anti-oxidation activity in AD model. From the oxidative-stress etiological theory of AD, we administrated scopolamine to stimulate AD, with chemical injuries to cholinergic nerve cells [20,21]. We measured SOD, MDA and GSH-PX levels to assess the potential of three levels of **C7** (low, medium, high) anti-oxidation activity in AD [22]. Piracetam, a classical AchE inhibitor for AD, was selected as a positive control, because many former studies of AD involving similar models (scopolamine administration) adopted piracetam as a positive control [23,24]. From Figure 3A, we can see the SOD level was significantly lower in model mice than in non-model mice (*p* < 0.01) and significantly higher in mice treated with piracetam or **C7** than in model mice (*p* < 0.01). From Figure 3B we can also see MDA level was significantly higher in model mice than in non-model mice (*p* < 0.01) and piracetam and **C7** can significantly down-regulate the MDA level in model mice (*p* < 0.01). Figure 3C shows levels of GSH-PX. GSH-PX level was significantly lower in model mice than in non-model mice (*p* < 0.01), and was significantly higher with piracetam or **C7** treatment mice than in model mice (*p* < 0.01).

The increase in brain oxidative damage might be facilitated by a failure of antioxidant defense mechanisms. Antioxidant enzymes that are important in preventing an excessive accumulation of ROS include Cu/Zn- and Mn-SOD, GSH-Px, GSSG-R [25,26]. Membrane lipids present in subcellular organelles are highly susceptible to free radical damage. Malondialdehyde (MDA) is a by-product of lipid peroxidation induced by free radicals and is also widely used as a biomarker of oxidative stress [27].

We measured SOD, MDA and GSH-PX levels to assess the potential of three levels of **C7** (low, medium, high) against oxidative damage in AD [20,22]. The results showed **C7** was a potential antioxidant in Alzheimer’s models *in vivo*.

## 3. Experimental

### 3.1. General

All synthetic reagents were from Shanghai Jingchun Reagents Co. (Shanghai, China); SOD, MDA and GSH-PX testing kits were from Nanjing Jiancheng Bioengineering Institute (Nanjing, China).

### 3.2. Chemistry

Synthesis of Hydroxyl-substituted Chalcone Analogs

**C1**–**3** were successfully synthesized via Claisen-Schmidt condensation using NaOH as a catalyst. Substituted benzaldehyde (0.2 mol), substituted acetophenone (0.22 mol) and NaOH (30 g) were dissolved in a mixed solvent of H_2_O (100 mL) and ethanol (50 mL) in a 250-mL pear-shaped flask. The mixtures were stirred for 1–24 h in room temperature. Then, the mixtures turned into yellow or a dark red sticky liquids and saturated hydrochloric acid was added until the pH value was about 1. Then yellow solids were obtained and the products were purified by recrystallization from acetone and H_2_O. The products were obtained in yields of 60–75%. For analytical data see Table 1.

**C4**–**7** wer also successfully synthesized via Claisen-Schmidt condensation using pyperidine as a catalyst. A 250-mL pear-shaped flask equipped with a effective magnetic stirring and a reflux condenser, contaning a mixture of hydroxyl-substituted benzaldehyde (0.2 mol), 2-hydroxylacetophenone (0.22 mol) and piperidine (10 mL), was put into an oil bath (160 °C). After 10 min, the mixture turned into a dark red sticky liquid and was poured, with three ethanol absolute-rinses, into a 500-mL beaker with sufficient 10% NaOH in an ice-bath. With vigorous stirring, the mixture turned into an orange-red, non-sticky and uniform solution. Then, saturated hydrochloric acid was added until the pH value reached 1–2, and absolute ethanol was added to form a mixture of a turbid yellow solution and a dark-brown floating oily substance. The mixture was cooled and kept at 4–5 °C for one day. The yellow solid crystallized from the solution was filtered with adequate ethanol-absolute washing until it appeared as bright-yellow crystals and was dried. The products were obtained in yields of 60–75%. For analytical data see Table 1.

### 3.3. Pharmacology

#### 3.3.1. Tests of Activity of ***C1–7*** in Scavenging DPPH-free Radicals

The antioxidant activity of the **C1–7** on DPPH-free radicals was estimated according to the procedure reported by Molyneux, Goupy *et al.* [28,29]. **C1**–**7** and Vc (positive control) were prepared as ethanol-absolute solutions of 0.19, 0.38, 0.5, 1.5, 3, 6, and 12 mmol/L. All absorption values were measured in 517 nm. The antioxidant activity (AA) of the sample was expressed as percentage disappearance of DPPH:

AA (%) = [1 − (A _control_ − A _sample_)/A _blank]_ × 100%

where A _blank_ is absorption values of DPPH in the presence of water instead of compounds.

#### 3.3.2. Tests of Activity of ***C1–7*** in Scavenging OH Free Radicals

The antioxidant activity of the **C1**–**7** on OH free radicals was estimated according to the procedure reported by Yu *et al.* [30]. 0.1% H_2_O_2_ (29.5 mmol/L, calibrated by potassium permanganate titration), 7.5 mmol/L ferrous sulfate (freshly prepared), 7.5 mmol/L phen (dissolved in ethanol), 0.1 mmol/L acetic acid (pH = 3.0), **C1**–**7** and **Vc** (positive control) were prepared as ethanol-absolute solutions of 0.19, 0.38, 0.75, 1.5, 3, 6, 12 mmol/L. All absorption values were measured in 536 nm. The antioxidant activity (AA) of the sample was expressed as percentage disappearance of OH:

AA (%) = [(A _sample_ − A _control_) − (A _injured_ − A _blank_)]/(A _uninjured_ − A _injured_) × 100%

#### 3.3.3. Tests of C7 on SOD, MDA and GSH-PX Levels on Scopolamine-Induced Alzheimer’s Models

We obtained 60 Kunming mice, weighing 22–25 g, from the barrier unit at the Laboratory Animal Center of Southern Medical University (Guangzhou, China). Animals were subjected to alternate 12 h periods of dark and light (lights on at 6:00 to 18:00) with temperature about 25 °C and humidity 40%–60%. Mice were randomly divided into six groups (10 rats in each group): control group, scopolamine group (model group), piracetam group, different dose of **C7** group (low dose, medium dose, high dose). Mice in piracetam group and **C7** group were intragastrically (i.g.) administered piracetam (500 mg/kg), **C7** (125 mg/kg, 250 mg/kg, 500 mg/kg), respectively, for 14 consecutive days. Mice in the control and model groups were treated similarly with corresponding volumes of saline. All mice except control group received scopolamine (5 mg/kg by intraperitoneal injection) after the last treatment of drugs or saline on day 14. Thirty min later, animals were sacrificed, and whole brain tissues were removed. Homogenates were made of 10% (w/v) in cold normal saline, and supernatants were collected immediately after 12,000 r/min centrifugation for 15 min and stored at −20 °C following the instructions of Nanjing Jiancheng Bioengineering Institute.

### 3.4. Statistical Analysis

Data are shown as mean+/−SD, and analysis involved use of SPSS 10.0 (SPSS Inc., Chicago, IL, USA) by one-way ANOVA followed by SNK-LSD test.

## 4. Conclusions

A growing body of evidence indicates that increased oxidative stress resulting from free radical damage to cellular functions is associated with a number of age-related disorders including atherosclerosis and arthritis. In recent years, considerable data have indicated that free radicals play a significant role in the pathogenesis of neurodegenerative disorders and promote the progression of AD [24]. So anti-free radical and anti-oxidation could be a potential therapeutic target to prevent and treat AD.

**C7**, a novel chalcone analoge was confirmed to be highly potent in scavenging DPPH and OH free radicals *in vitro*. Tests of anti-free radical activity in response to oxidative stress in mice revealed that **C7** could elevate glutathione peroxidase (GSH-PX) and super oxide dismutase (SOD) levels and lower malonaldehyde (MDA) level in a free-radical-injured scopolamine-induced Alzheimer’s model. Further behavioral tests would be applied to examine the **C7** effect on AD in future studies. The preliminary study of structure-activity relationships of **C7** indicated that its activity maybe be related to its structure which comprised both a 4-hydroxyl substituted chalcone and 3,5 dimethoxy-substitution. In summary, **C7**, a novel hydroxyl-substituted chalcone analogs possesses ree radical scavenging properties *in vitro* and anti-oxidation properties in a free radical–injury Alzheimer’s model *in vivo.* It can be a preclinical anti-AD drug candidate for the next steps of research.
